# Vaccination with BNT162b2 reduces transmission of SARS-CoV-2 to household contacts in Israel

**DOI:** 10.1126/science.abl4292

**Published:** 2022-01-27

**Authors:** Ottavia Prunas, Joshua L. Warren, Forrest W. Crawford, Sivan Gazit, Tal Patalon, Daniel M. Weinberger, Virginia E. Pitzer

**Affiliations:** ^1^ Department of Epidemiology of Microbial Diseases, Yale School of Public Health, Yale University, New Haven, CT, USA.; ^2^ Public Health Modeling Unit, Yale School of Public Health, Yale University, New Haven, CT, USA.; ^3^ Department of Biostatistics, Yale School of Public Health, Yale University, New Haven, CT, USA.; ^4^ Department of Statistics and Data Science, Yale School of Public Health, Yale University, New Haven, CT, USA.; ^5^ Department of Ecology and Evolutionary Biology, Yale School of Public Health, Yale University, New Haven, CT, USA.; ^6^ Yale School of Management, Yale University, New Haven, CT, USA.; ^7^ Maccabi Institute for Research and Innovation, Maccabi Healthcare Services, Tel Aviv, Israel.

## Abstract

The individual-level effectiveness of vaccines against COVID-19 is well established. However, few studies have examined vaccine effectiveness against transmission. We used a chain binomial model to estimate the effectiveness of vaccination with BNT162b2 (Pfizer-BioNTech mRNA-based vaccine) against household transmission of SARS-CoV-2 in Israel before and after the Delta variant emerged. Vaccination reduced susceptibility to infection by 89.4% [95% confidence interval (CI): 88.7%, 90.0%], whereas vaccine effectiveness against infectiousness given infection was 23.0% (95% CI: −11.3%, 46.7%) during days 10 to 90 after the second dose before June 1, 2021. Total vaccine effectiveness was 91.8% (95% CI: 88.1%, 94.3%). However, vaccine effectiveness is reduced over time as a result of the combined effect of waning of immunity and the emergence of the Delta variant.

The COVID-19 pandemic has led to unprecedented disruptions worldwide. The rapid development and deployment of vaccines against SARS-CoV-2 has provided an opportunity to control the outbreak in populations with access to vaccination. Multiple vaccines against SARS-CoV-2 effectively prevent clinical disease and reduce disease severity in those who do become infected (*1*–*3*). This direct protection against disease is critical. However, additional population-level benefits can be derived if vaccines also reduce transmission of the virus, thereby providing protection to those who are still vulnerable to infection (*1**, **4*).

To date, there is little direct real-world evidence about the effects of vaccination on SARS-CoV-2 transmission. A few studies have investigated the reduction in transmission in households and among healthcare workers (*3**, **5**, **6*). Other studies have found indirect evidence for a likely effect of the vaccine on transmission by demonstrating reduced viral load in the upper respiratory tract of infected individuals (*7*–*11*). These studies have mostly focused on the period when the Alpha variant was the dominant strain and have not examined the effects on transmission following emergence of the Delta variant (*12*).

Households are an ideal setting for evaluating transmission of the virus and the effects of vaccination as a result of the high secondary attack rate (SAR) among household members (*3**, **14*). Detailed data on household structure and timing of infections can be used to quantify the risk of transmission. We aimed to assess the effectiveness of vaccination with BNT162b2 (Pfizer-BioNTech mRNA-based vaccine) against susceptibility to infection and against infectiousness given infection with SARS-CoV-2, comparing the pre- and post-Delta periods. We accomplished this by means of a chain binomial model, which is a common approach for reconstruction of transmission in household settings (*14*), applied to data from the second largest healthcare organization in Israel. The rapid and early rollout of mass vaccination in Israel provides a notable opportunity to evaluate the effectiveness of vaccination against transmission.

We used data from the centralized database of Maccabi Healthcare Services (MHS), which captures all information on the demographics and healthcare-related interactions of members. MHS is a nationwide 2.5-million-member, state-mandated, not-for-profit healthcare delivery organization in Israel, representing a quarter of the Israeli population. The full dataset, covering the period from June 1, 2020 to July 28, 2021, included information on 2,472,502 individuals from 1,327,647 households. Among these, 1,471,386 individuals received two doses of BNT162b2 as of July 28, 2021 (before the widespread introduction of booster doses). There were 202,298 detected infections caused by SARS-CoV-2 (8.2% of the total population), with 6,483 infections in fully vaccinated individuals at the time of their polymerase chain reaction (PCR) test date and 186,975 infections in unvaccinated individuals (unadjusted risk ratio = 6.6%) (table S1 and fig. S1).

Most of the households (60% of the total) had a single household member; this individual was infected in 62,295 (7.8%) of the 797,170 households. Information on the number of households and proportion of infections occurring in households of varying size can be found in table S2. The naïve SAR, on the basis of the vaccination status of the “index case” (defined as the first person to test positive in a household), was lower when the index case was vaccinated during the pre-Delta period (table S3).

We used a chain binomial model for household transmission to estimate how the probability of infection per day depended on the characteristics of susceptible individuals and their household contacts (*14**, **15*). An individual’s infection probability is modeled as the risk of escaping infection from the community and any/all infectious household members on each day of exposure (see materials and methods). We used multiple imputation to generate latent data for when a person with a positive PCR test was infected and infectious. This was accomplished by using random samples from three different Gamma distributions representing the delay between onset of infectiousness and the date of the PCR test, the date of infection and the onset of infectiousness (i.e., the latent period), and the onset of infectiousness to the end of infectiousness (i.e., the infectious period) (Fig. 1 and table S4; materials and methods). We performed sensitivity analyses to confirm the robustness of our results to variability in the delay distributions and performed a simulation study to validate our approach (figs. S2 to S5 and tables S5 to S7; materials and methods).

**Fig. 1.**
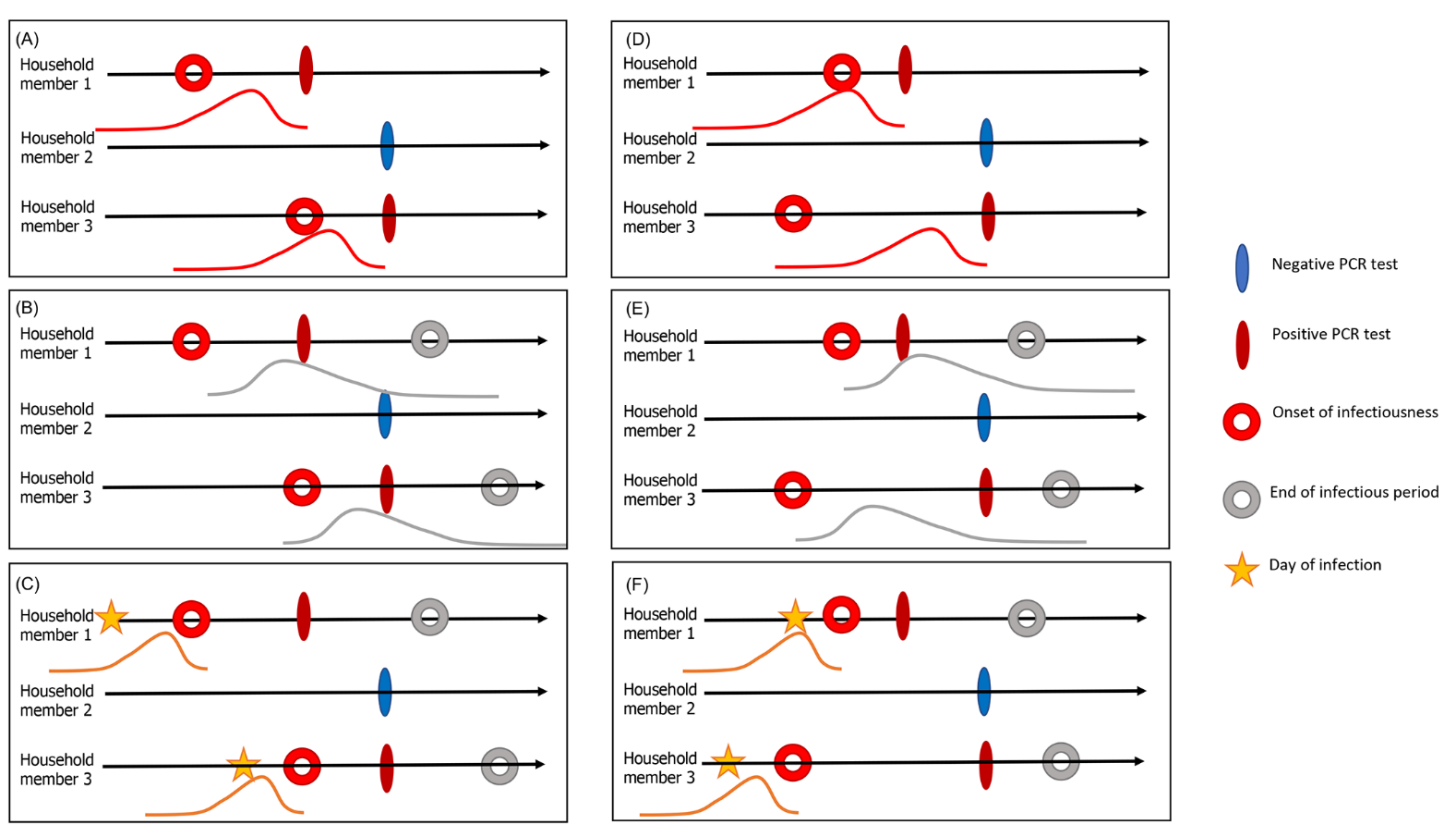
Schematic representation of the multiple imputation process for an example household. Each infected household member is associated with: (**A **and** D**) a distribution for time from onset of infectiousness to testing; (**B **and** E**) a distribution for the infectious period; and (**C **and** F**) a distribution for the latent period to infer the time of infection. The filled ovals represent observed events, and the circles and stars represent unobserved events in the infection timeline. Panels (A to C) and (D to F) represent two possible sample sets from the delay distributions, each with a different index case, who is not necessarily the first person to test positive in the household. We generated 100 samples of the latent data for each infected individual.

The pairwise daily probability of infection from the community and from each infected household member was modeled as a function of the time-varying number of SARS-CoV-2-positive individuals in the population, the characteristics of the susceptible individual (including age and vaccination status), and the vaccination status of their household contacts. We considered four categories of vaccination: unvaccinated; ≥10 days from dose 1 to <10 days from dose 2; ≥10 days to <90 days from dose 2; and ≥90 days from dose 2 to account for partial vaccination, full vaccination, and waning of vaccine-induced immunity. Vaccine effectiveness against susceptibility to infection was estimated from the coefficient of the susceptible individual’s vaccination status, whereas vaccine effectiveness against infectiousness given infection was estimated from the coefficient of the vaccination status of each infectious household member. To determine the impact of the Delta variant, we allowed the vaccine effects to vary before and after June 1, 2021 (i.e., the pre- and post-Delta period, respectively). We estimated the effects by averaging over 100 draws from the delay distributions used in the multiple imputation process; the variance of the estimates across these 100 draws was estimated with the law of total variance (figs. S6 to S8).

For the period before June 1, 2021 (before emergence of the Delta variant), receipt of two doses of the vaccine was associated with a vaccine effectiveness against susceptibility to infection (*VE_S_*) of 89.4% [95% confidence interval (CI): 88.7%, 90.0%] within 10 to 90 days of receiving the second dose, and 58.3% (95% CI: 45.8%, 67.9%) more than 90 days after receiving the second dose. The vaccine effectiveness against infectiousness given infection (*VE_I_*) was 23.0% (95% CI: −11.3%, 46.7%) within 10 to 90 days and 6.9% (95% CI: −124.8%, 61.4%) more than 90 days after the second dose (Table 1). The total vaccine effectiveness (*VE_T_*), which combines the reduction in the risk of infection and the risk of infectiousness given infection among vaccinated individuals, was estimated to be 91.8% (95% CI: 88.1%, 94.3%) within 10 to 90 days, and 61.1% (95% CI: 5.2%, 84.1%) more than 90 days after the second dose. Evidence of waning protection following vaccination was apparent for the ≥90-day time period after the second dose for all vaccine effects (Table 1).

**Table 1.** Vaccine effectiveness against susceptibility to infection (*VE_S_*); vaccine effectiveness against infectiousness given infection (*VE_I_*); total vaccine effectiveness (*VE_T_*) at different time ranges since vaccination, both before and after the emergence of the Delta variant (June 1, 2021).

**Vaccine effectiveness measure**	**Time since vaccination**	**Estimate pre-Delta** **[95% confidence interval]**	**Estimate post-Delta** **[95% confidence interval]**
*Vaccine effectiveness against susceptibility to infection*			
$VE_{S1}$	≥10d dose 1 and <10d dose 2	62.7% [61.5%, 63.8%]	72.1% [66.7%, 75.6%]
$VE_{S2}$	≥10d dose 2 and <90d dose 2	89.4% [88.7%, 90.0%]	72.0% [65.9%, 77.0%]
$VE_{S3}$	≥90d dose 2	58.3% [45.8%, 67.9%]	40.2% [37.6%, 42.6%]
*Vaccine effectiveness against infectiousness given infection*			
$VE_{I1}$	≥10d dose 1 and <10d dose 2	−15.9% [−27.9%, −5.0%]	38.3% [−24.2%, 69.3%]
$VE_{I2}$	≥10d dose 2 and <90d dose 2	23.0% [−11.3%, 46.7%]	−27.9% [−248.9%, 53.1%]
$VE_{I3}$	≥90d dose 2	6.9% [−124.8%, 61.4%]	−27.9% [−53.7%, −6.5%]
*Total vaccine effectiveness*			
$VE_{T1}$	≥10d dose 1 and <10d dose 2	56.8% [52.2%, 60.9%]	82.8% [64.8%, 91.6%]
$VE_{T2}$	≥10d dose 2 and <90d dose 2	91.8% [88.1%, 94.3%]	65.6% [4.9%, 87.6%]
$VE_{T3}$	≥90d dose 2	61.1% [5.2%, 84.1%]	24.2% [9.0%, 36.9%]

d, days.

Following the emergence of the Delta variant, we observed a marked reduction in the vaccine effectiveness against susceptibility to infection compared with the pre-Delta period. During this period, the *VE_S_* was 72.0% (95% CI: 65.9%, 77.0%) within 10 to 90 days and 40.2% (95% CI: 37.6%, 42.6%) more than 90 days after the second dose. A similar finding was observed for total vaccine effectiveness: *VE_T_* = 65.6% (95% CI: 4.9%, 87.6%) within 10 to 90 days and 24.2% (95% CI: 9.0%, 36.9%) more than 90 days after the second dose. There was a high degree of uncertainty in the estimates of vaccine effectiveness against infectiousness given infection during the Delta period (Table 1). Allowing for differences in vaccine effectiveness for the post-Delta period improved the model fit, based on a comparison of the Akaike Information Criteria (figs. S9 and S10).

We further analyzed the effect of vaccination on infectiousness given infection when restricting our data to the susceptible unvaccinated population (i.e., children <12 years of age). We observed a larger reduction in risk for children exposed to a vaccinated versus unvaccinated infectious household member, with *VE_I_* = 41.0% (95% CI: −13.7%, 69.4%) between 10 to 90 days from receiving the second dose (table S8). The corresponding vaccine effect during the Delta period was not significantly different from zero.

The probability of transmission per day from an infected household member to a susceptible adult during the pre-Delta period was 0.021 (95% CI: 0.020, 0.021), leading to a SAR of 0.10 (95% CI: 0.09, 0.10) (table S9; materials and methods). The risk of transmission from an infectious household member was ~100 times as high as that of the average risk of infection from the community. During the period when the Delta variant was dominant, there was no meaningful increase in the household transmission probability, whereas there was an increase in the risk of infection from the community (*RR *= 1.13; 95% CI: 1.09, 1.16) (table S9). Children <12 years old had a lower risk of infection from both the community and an infectious household member, whereas adults 40 to 64 years and ≥65 years of age had a lower risk of infection from the community but a higher risk of infection within the household compared with individuals aged 12 to 39 years (table S9). In a sensitivity analysis, we found that children were slightly less infectious than adults (see SM).

To date, there is limited evidence with which to compare our estimates of vaccine effectiveness against infectiousness and transmission. A study of over 550,000 households in England showed that vaccination with both the ChAdOx1 nCoV-19 and BNT162b2 vaccines reduced the odds of transmission from a vaccinated and infected household member by 40 to 50% compared with unvaccinated index cases (*1**, **3*). A similar study in Denmark estimated the reduction in transmission to be 42% during the Delta period (*16*). In previous studies, the index case in each household was defined as the earliest case of laboratory-confirmed COVID-19, by diagnosis date, and all secondary infections in the household were attributed to the index case (*3*). By contrast, by inferring the date of infection we do not assume that the index case in the household was necessarily the first individual to be diagnosed, and we account for the risk of transmission from other infected household members and from the community. With our approach, we show a lower and uncertain reduction in infectiousness given infection, compared with simpler methods (*16**–**18*). A comparable statistical approach was used in another study in Israel, where they actively followed and tested household members with confirmed cases and observed a notably higher reduction in infectivity, though with large uncertainty; however, the study was limited to healthcare workers, who normally represent a younger and healthier population, thereby potentially leading to a stronger vaccine effect (*6*). Other studies investigating the reduction in infection risk among household members of vaccinated versus unvaccinated healthcare workers were conducted in Scotland and Finland, providing indirect evidence of a lower risk of infection among household contacts of vaccinated individuals (*1**, **5**, **19*).

Our analyses suggest that before emergence of the Delta variant, breakthrough cases among vaccinated individuals had slightly reduced infectiousness compared with unvaccinated cases. However, both waning of vaccine-induced immunity and the emergence of the Delta variant were associated with a reduction in the *VE_I_*. These results are in agreement with recent findings in a UK study, where the SAR was similar for vaccinated and unvaccinated index cases infected with the Delta variant (*12*). However, vaccination still reduces the risk of transmission by providing protection against susceptibility to infection, even if this effect is reduced over time because of both waning immunity and the Delta variant, as highlighted in real-world settings (*12**, **20**, **21*).

This study has several important limitations. We did not have information on the true infection times (and duration of infectiousness) of infected household members. To overcome this limitation, we sampled from three delay distributions parameterized from the literature to determine the potential infection status of each individual through time. Our approach is suboptimal, however, because it was not computationally feasible to estimate the parameters of the delay distributions conditional on the observed data, e.g., by means of an expectation-maximization or Markov chain Monte Carlo approach. As a result, parameter estimates do not reflect uncertainty in the delay distribution parameters. This could lead to artificially narrow confidence intervals for some parameters. In addition, the $VE_{I}$ estimates were dependent upon the specification of the time from onset of infectiousness to testing (fig. S5). Also, individuals who were infected but did not receive a SARS-CoV-2 test would be misclassified in our dataset. This is likely to have only a minor effect on our estimates, though the *VE_I_* could be underestimated if the probability of detection per day is low (see SM, tables S6 and S7). We estimated a negative *VE_I_* in partially vaccinated cases, suggesting possible sources of bias in our analysis (e.g., partially vaccinated individuals may be less likely to isolate at the first sign of symptoms). This effect is mitigated during the post-Delta period (Table 1). Controlling for the age of infectious individuals did not resolve the potential bias (table S10). Finally, our results do not include the period when the Omicron variant has become dominant, although recent findings suggest that SARs among unvaccinated household members are comparable to the Delta variant (*22*).

Vaccination can prevent transmission by both providing protection against infection (including asymptomatic infections) and reducing the infectiousness of vaccinated individuals who do become infected. Neither of these are typically directly measured in vaccine trials. By analyzing data on confirmed SARS-CoV-2 infections among household members in Israel, we provide measures of effectiveness of BNT162b2 against susceptibility to infection and against infectiousness given infection. Our results show evidence of a slight reduction in the infectiousness of vaccinated individuals who become infected in addition to protection against susceptibility to infection, leading to an overall reduction in the risk of transmission. However, the ability of vaccination to prevent transmission is reduced over time because of waning of vaccine-induced immunity and lower effectiveness against the Delta variant. It is highly unlikely that population-level transmission of SARS-CoV-2 can be eliminated through vaccination alone.

## Supplementary Materials

### This PDF file includes:

Materials and Methods Figs. S1 to S10 Tables S1 to S10 References (*24*–*30*)

### Other Supplementary Material for this manuscript includes the following:

MDAR Reproducibility Checklist

## References

[1] A. Richterman, E. A. Meyerowitz, M. Cevik, Indirect Protection by Reducing Transmission: Ending the Pandemic with SARS-CoV-2 Vaccination. *Open Forum Infect. Dis.* **9**, ofab259 (2021). 10.1093/ofid/ofab259 PMC8194790 35071679

[2] N. Dagan, N. Barda, E. Kepten, O. Miron, S. Perchik, M. A. Katz, M. A. Hernán, M. Lipsitch, B. Reis, R. D. Balicer, BNT162b2 mRNA Covid-19 Vaccine in a Nationwide Mass Vaccination Setting. N. Engl. J. Med. 384, 1412–1423 (2021). 10.1056/NEJMoa2101765 33626250 PMC7944975

[3] R. J. Harris, J. A. Hall, A. Zaidi, N. J. Andrews, J. K. Dunbar, G. Dabrera, Impact of vaccination on household transmission of SARS-COV-2 in England 2021; Available from: https://khub.net/documents/135939561/390853656/Impact+of+vaccination+on+household+transmission+of+SARS-COV-2+in+England.pdf/35bf4bb1-6ade-d3eb-a39e-9c9b25a8122a. 10.1056/NEJMc2107717 PMC8262621 34161702

[4] Z. J. Madewell, Y. Yang, I. M. Longini Jr., M. E. Halloran, N. E. Dean, Household Transmission of SARS-CoV-2: A Systematic Review and Meta-analysis. JAMA Netw. Open 3, e2031756 (2020). 10.1001/jamanetworkopen.2020.31756 33315116 PMC7737089

[5] V. Shah ., Effect of vaccination on transmission of COVID-19: an observational study in healthcare workers and their households. medRxiv, 2021: p. 2021.03.11.21253275.

[6] M. Layan, M. Gilboa, T. Gonen, M. Goldenfeld, L. Meltzer, A. Andronico, N. Hozé, S. Cauchemez, G. Regev-Yochay, Impact of BNT162b2 vaccination and isolation on SARS-CoV-2 transmission in Israeli households: an observational study. medRxiv, 2021. 10.1093/aje/kwac042 PMC8903452 35238329

[7] M. Levine-Tiefenbrun, I. Yelin, R. Katz, E. Herzel, Z. Golan, L. Schreiber, T. Wolf, V. Nadler, A. Ben-Tov, J. Kuint, S. Gazit, T. Patalon, G. Chodick, R. Kishony, Initial report of decreased SARS-CoV-2 viral load after inoculation with the BNT162b2 vaccine. Nat. Med. 27, 790–792 (2021). 10.1038/s41591-021-01316-7 33782619

[8] X. Qiu, A. I. Nergiz, A. E. Maraolo, I. I. Bogoch, N. Low, M. Cevik, Defining the role of asymptomatic and pre-symptomatic SARS-CoV-2 transmission–a living systematic review. *Clin. Microbiol. Infect**.* (2021). 10.1016/j.cmi.2021.01.011 PMC7825872 33484843

[9] M. Marks, P. Millat-Martinez, D. Ouchi, C. H. Roberts, A. Alemany, M. Corbacho-Monné, M. Ubals, A. Tobias, C. Tebé, E. Ballana, Q. Bassat, B. Baro, M. Vall-Mayans, C. G-Beiras, N. Prat, J. Ara, B. Clotet, O. Mitjà, Transmission of COVID-19 in 282 clusters in Catalonia, Spain: A cohort study. Lancet Infect. Dis. 21, 629–636 (2021). 10.1016/S1473-3099(20)30985-3 33545090 PMC7906723

[10] E. Petter ., Initial real world evidence for lower viral load of individuals who have been vaccinated by BNT162b2. medRxiv, 2021: p. 2021.02.08.21251329.

[11] F. P. Lyngse ., Association between SARS-CoV-2 Transmission Risk, Viral Load, and Age: A Nationwide Study in Danish Households. medRxiv, 2021: p. 2021.02.28.21252608.

[12] A. Singanayagam *et al*., *Community Transmission and Viral Load Kinetics of SARS-CoV-2 Delta (B. 1.617. 2) Variant in Vaccinated and Unvaccinated Individuals**.* 2021. 10.1016/S1473-3099(21)00648-4 PMC8554486 34756186

[13] V. E. Pitzer, T. Cohen, Household studies provide key insights on the transmission of, and susceptibility to, SARS-CoV-2. Lancet Infect. Dis. 20, 1103–1104 (2020). 10.1016/S1473-3099(20)30514-4 32562602 PMC7832097

[14] A. H. Rampey Jr., I. M. Longini Jr., M. Haber, A. S. Monto, A discrete-time model for the statistical analysis of infectious disease incidence data. Biometrics 48, 117–128 (1992). 10.2307/2532743 1316178

[15] Y. Yang, I. M. Longini Jr., M. E. Halloran, Design and evaluation of prophylactic interventions using infectious disease incidence data from close contact groups. Appl. Stat. 55, 317–330 (2006). 10.1111/j.1467-9876.2006.00539.x 22457545 PMC3312606

[16] F. P. Lyngse ., Effect of Vaccination on Household Transmission of SARS-CoV-2 Delta VOC. medRxiv, 2022: p. 2022.01.06.22268841. 10.1038/s41467-022-31494-y PMC9244879 35773247

[17] D. W. Eyre, D. Taylor, M. Purver, D. Chapman, T. Fowler, K. B. Pouwels, A. S. Walker, T. E. A. Peto, Effect of Covid-19 Vaccination on Transmission of Alpha and Delta Variants. *N. Engl. J. Med.* NEJMoa2116597 (2022). 10.1056/NEJMoa2116597 PMC8757571 34986294

[18] R. J. Harris ., Impact of vaccination on household transmission of SARS-COV-2 in England. medRxiv, 2021. 10.1056/NEJMc2107717 PMC8262621 34161702

[19] J. Salo ., The indirect effect of mRNA-based Covid-19 vaccination on unvaccinated household members. medRxiv, 2021: p. 2021.05.27.21257896. 10.1038/s41467-022-28825-4 PMC8897446 35246536

[20] T. Patalon ., Short Term Reduction in the Odds of Testing Positive for SARS-CoV-2; a Comparison Between Two Doses and Three doses of the BNT162b2 Vaccine. medRxiv, 2021.

[21] B. Mizrahi ., Correlation of SARS-CoV-2 breakthrough infections to time-from-vaccine; Preliminary study. MedRxiv, 2021. 10.1038/s41467-021-26672-3 PMC8569006 34737312

[22] F. P. Lyngse ., SARS-CoV-2 Omicron VOC Transmission in Danish Households. medRxiv, 2021.

[23] O. W. Prunas, J. L. Warren, F. W. Crawford, S. Gazit, T. Patalon, D. M. Weinberger, V. E. Pitzer, Data from: “Vaccination with BNT 162b2 reduces transmission of SARS-VoC-2 to household Contacts in Israel”. Zenodo, 2021. DOI: 10.5281/zenodo.5670699 PMC9261115 35084937

[24] E. J. Haas, F. J. Angulo, J. M. McLaughlin, E. Anis, S. R. Singer, F. Khan, N. Brooks, M. Smaja, G. Mircus, K. Pan, J. Southern, D. L. Swerdlow, L. Jodar, Y. Levy, S. Alroy-Preis, Impact and effectiveness of mRNA BNT162b2 vaccine against SARS-CoV-2 infections and COVID-19 cases, hospitalisations, and deaths following a nationwide vaccination campaign in Israel: An observational study using national surveillance data. Lancet 397, 1819–1829 (2021). 10.1016/S0140-6736(21)00947-8 33964222 PMC8099315

[25] E. Leshem, A. Wilder-Smith, COVID-19 vaccine impact in Israel and a way out of the pandemic. Lancet 397, 1783–1785 (2021). 10.1016/S0140-6736(21)01018-7 33964221 PMC8099312

[26] N. G. Davies, A. J. Kucharski, R. M. Eggo, A. Gimma, W. J. Edmunds, T. Jombart, K. O’Reilly, A. Endo, J. Hellewell, E. S. Nightingale, B. J. Quilty, C. I. Jarvis, T. W. Russell, P. Klepac, N. I. Bosse, S. Funk, S. Abbott, G. F. Medley, H. Gibbs, C. A. B. Pearson, S. Flasche, M. Jit, S. Clifford, K. Prem, C. Diamond, J. Emery, A. K. Deol, S. R. Procter, K. van Zandvoort, Y. F. Sun, J. D. Munday, A. Rosello, M. Auzenbergs, G. Knight, R. M. G. J. Houben, Y. Liu, Centre for the Mathematical Modelling of Infectious Diseases COVID-19 working group, Effects of non-pharmaceutical interventions on COVID-19 cases, deaths, and demand for hospital services in the UK: A modelling study. Lancet Public Health 5, e375–e385 (2020). 10.1016/S2468-2667(20)30133-X 32502389 PMC7266572

[27] Y. M. Bar-On, Y. Goldberg, M. Mandel, O. Bodenheimer, L. Freedman, N. Kalkstein, B. Mizrahi, S. Alroy-Preis, N. Ash, R. Milo, A. Huppert, Protection of BNT162b2 vaccine booster against Covid-19 in Israel. N. Engl. J. Med. 385, 1393–1400 (2021). 10.1056/NEJMoa2114255 34525275 PMC8461568

[28] J. E. Dennis Jr., R. B. Schnabel, *Numerical methods for unconstrained optimization and nonlinear equations*. 1996: SIAM.

[29] R. B. Dessau, C. B. Pipper, Ugeskr. Laeger 170, 328–330 (2008) [“R”—project for statistical computing]. 18252159

[30] Q.-L. Jing, M.-J. Liu, Z.-B. Zhang, L.-Q. Fang, J. Yuan, A.-R. Zhang, N. E. Dean, L. Luo, M.-M. Ma, I. Longini, E. Kenah, Y. Lu, Y. Ma, N. Jalali, Z.-C. Yang, Y. Yang, Household secondary attack rate of COVID-19 and associated determinants in Guangzhou, China: A retrospective cohort study. Lancet Infect. Dis. 20, 1141–1150 (2020). 10.1016/S1473-3099(20)30471-0 32562601 PMC7529929

