# Supplementary material for: Vaccination with BNT162b2 reduces transmission of SARS-CoV-2 to household contacts in Israel
Source: Science. 2022 Jan 27;375(6585):eabl4292. doi: 10.1126/science.abl4292 (PMC9261115; doi:10.1126/science.abl4292)
Supplement: Supplementary file 1 — Materials and Methods Figs. S1 to S10 Tables S1 to S10 References (24–30) [file science.abl4292_sm.pdf]

Science
AAAS

Supplementary Materials for

Vaccination with BNT162b2 reduces transmission of SARS-CoV-2 to household contacts in Israel

Ottavia Prunas *et al.*

Corresponding author: Ottavia Prunas, ottavia.prunas@yale.edu

DOI: 10.1126/science.abl4292

The PDF file includes:

Materials and Methods
Figs. S1 to S10
Tables S1 to S10
References

Other Supplementary Material for this manuscript includes the following:

MDAR Reproducibility Checklist

Materials and Methods

Setting

Vaccination in Israel began on December 20, 2020, mainly using the BioNTech-Pfizer BNT162b2 vaccine, with a few individuals receiving the vaccine earlier. The vaccination campaign first targeted high-risk individuals, including those 60 years of age and older, medical personnel, workers at nursing homes, and individuals with comorbidities. After this phase, which lasted until January 21, 2021, age restrictions were lowered. By February 6, 2021, every Israeli citizen above 16 years old was eligible for the vaccine [11]. By the beginning of April 2021, 61% of the population had received at least one dose of the BNT162b2 vaccine [25].

The vaccination roll-out coincided with Israel's third and largest wave of SARS-CoV-2 registered cases [26] due to the rise of the Alpha variant, representing approximately 90% of sequenced isolates [7] (Fig. S1). Consequently, a third national lockdown was issued in Israel starting December 24, 2020, with more severe restrictions (e.g., school closures) issued starting January 8, 2021. These restrictions were progressively lifted starting February 7, 2021. By the beginning of the summer (mid-June 2021), a new wave driven by the Delta variant caused a rapid increase in number of cases, many in vaccinated individuals. This prompted the introduction of a national third-dose (booster) vaccination campaign beginning with high-risk populations on July 12 and individuals 60 years of age and older on July 30, 2021.

Data sources

We used data from Maccabi Healthcare Services (MHS) centralized computerized database, which captures all information on members and their healthcare-related interactions (e.g., demographics, inpatient and outpatient visits, diagnoses, procedures). MHS is a nationwide 2.5-million-member state-mandated, not-for-profit healthcare delivery organization in Israel, representing a quarter of the Israeli population. The individual-level data for individuals and their household contacts include demographic information (i.e., age, sex), date of any polymerase chain reaction (PCR) tests for SARS-CoV-2 and the result(s) of the test (considering that all such tests of MHS members are recorded centrally), and date of receipt of the first and second doses of the vaccine (if received). Individuals were defined as unvaccinated if they had not received any doses of BNT162b2, partially vaccinated within 10 days from the first dose until 10 days after the second dose, and fully vaccinated if at least 10 days had passed since receiving the second dose of the vaccine. We further divided the fully vaccinated category into ≥10 days to <90 days and ≥90 days post-dose 2 to examine potential waning of vaccine-induced immunity.

We defined the study period as June 1, 2020 to July 28, 2021. All households with at least one positive PCR test before June 1, 2020 were excluded from the analysis (including both the infected individual and their household contacts), since viral testing was not widely available prior to this date.

Naïve secondary attack rate (SAR)

We computed the naïve SAR as the number of contacts who tested positive within 14 days of the index cases divided by the total number of household contacts (Table S3). For this analysis, we defined the "index case" as the first person to test positive in the household, and excluded 7,951 households in which there was more than one index case. We distinguished between four categories depending on the vaccination status of the index case: unvaccinated, partially vaccinated (i.e., ≥10 days from the first dose until <10 days after the second dose), fully vaccinated (≥10 days to <90 days and ≥90 days post-dose 2); we also differentiated between index cases occurring before versus after the emergence of the Delta variant (June 1, 2021).

Multiple imputation approach for inferring transmission rates

For each individual, we observed the date at which the viral test was performed and the outcome of the test, but we do not have data on date of infection. To infer transmission rates, it is necessary to estimate when each individual within a household was infected and the period when each person was infectious. We therefore used multiple imputation to generate missing data on when a person with a positive PCR test was infected and infectious. This was accomplished using random samples from three different Gamma distributions representing the delay between onset of infectiousness and the date of the PCR test (τ_{report}), between the date of infection and the onset of infectiousness (τ_{latent}), and the time from the onset of infectiousness to the end of infectiousness ($\tau_{infectious}$). The parameters of these distributions were derived from observational studies on the latent period, time from infection to testing (which is assumed to be similar to the incubation period), and the duration of infectiousness (Table S4) [27, 28]. For each infected individual, a random draw was taken from each of these distributions; this process was repeated 100 times. We refer to these Gamma distributions as the *delay distributions*.

For each person i in household h with a positive PCR test, let T_{ih} be the (imputed) time of infection in days since the beginning of the study: $T_{ih} = day_{ih}^{test} - \tau_{report,ih} - \tau_{latent,ih}$, with day_{ih}^{test} being the number of days after the start of the study until the positive PCR test date. We set the beginning date of the study as May 15, 2020, since we allow for infections occurring up to 17 days prior to the start of the data (i.e., June 1, 2020). If no infection occurred for person i, T_{ih} is censored and equal to the total number of days in the study (i.e., t_{max}). For the purposes of model fitting, we define Y_{iht} as a binary variable equal to zero for each day of the study up until the time of infection ($t = 1, \dots, T_{ih} - 1$), equal to one for T_{ih} (assuming the person is infected), and censored from that point onwards (i.e., that person is relevant only in terms of transmitting to other household members). For people who are never infected, Y_{iht} is equal to zero for all days of the study.

<u>Chain binomial model for household transmission</u>

We used a discrete-time chain binomial model [15] to estimate vaccine effectiveness against susceptibility to infection and against infectiousness given infection. We model the infection status of person *i* in household *h* on study day *t* (Y_{iht}) using conditionally independent Bernoulli distributions with corresponding probability of infection π_{iht}. We define the probability of infection on a given day as:

$$\pi_{iht} = 1 - (1 - p_{i0t}) \prod_{j=1, j \neq i}^{n_h} (1 - p_{ijt})^{d_{jt}} \qquad (1)$$

where n_h is the number of members in household *h*, p_{i0t} is the probability that person *i* in household *h* is infected by the community on study day *t* (i.e., community risk of infection), p_{ijt} is the probability that person *i* is infected by household member *j* (i.e., household risk of infection), and d_{jt} is an indicator of whether person *j* can transmit infection on day *t*. In other words, $d_{jt} = 1\{\text{person } j \text{ ever infected}\} * 1\{t \in (day_{hj}^{onset}, day_{hj}^{end})\}$, with $day_{hj}^{onset} = T_{hj} + \tau_{latent,jh}$ and $day_{hj}^{end} = day_{hj}^{onset} + \tau_{infectious,hj,}$ equal to the time of onset and end of infectiousness, respectively, for person *j*, and 1(.) representing the indicator function equal to one if the input condition is true and equal to zero otherwise.

The probability that individual *i* never tested positive for SARS-CoV-2 is given as

$$\prod_{t=1}^{t_{max}} (1 - \pi_{iht}) = \prod_{t=1}^{t_{max}} (1 - p_{i0t}) \prod_{j=1, j \neq i}^{n_h} (1 - p_{ijt})^{d_{jt}}$$

whereas the probability that individual *i* is infected on day t^* (before the end of the study) is given as

$$\pi_{iht}\prod_{t=1}^{t^*-1}(1-\pi_{iht}) =$$
$$\left\{1-(1-p_{i0t^*})\prod_{j=1,j\neq i}^{n_h}(1-p_{ijt^*})^{d_{jt^*}}\right\}\left\{\prod_{t=1}^{t^*-1}(1-p_{i0t})\prod_{j=1,j\neq i}^{n_h}\left(1-p_{ijt}\right)^{d_{jt}}\right\},$$

(i.e., the probability of not escaping infection at time t^* multiplied by the probability of escaping infection up to time (t^*-1)).

The likelihood function can be written in terms of the introduced binary variables such that:

$$\prod_{h=1}^{N}\prod_{i=1}^{n_h}\prod_{t=1}^{T_{ih}}P(Y_{iht}=y_{iht}|\pi_{iht}) = \prod_{h=1}^{N}\prod_{i=1}^{n_h}\prod_{t=1}^{T_{ih}}\pi_{iht}^{y_{iht}}(1-\pi_{iht})^{1-y_{iht}}$$

with *N* being the total number of households.

We define the individual- and time-specific community risk of infection using the log link function as:

$$\ln(p_{i0t}) = \delta_0 + \beta_1 vax_{1,it} + \beta_2 vax_{2,it} + \beta_3 vax_{3,it} + \gamma_1{}^{comm} age_{1,i} + \gamma_2{}^{comm} age_{2,i} + \gamma_3{}^{comm} age_{3,i} + \delta_1 cases_t \qquad (2)$$

where δ_0 is the baseline risk of infection from the community; $vax_{1,it}$ is a binary variable equal to one if ≥10 days has passed since person *i* received the first dose of the vaccine and <10 days from the second dose; $vax_{2,it}$ is a binary variable equal to one within 10-89 days since the second dose of the vaccine; $vax_{3,it}$ is a binary variable equal to one if ≥90 days have passed since the second dose of the vaccine; $age_{1,i}$ is a binary variable equal to one if the person is 0-11 years old at the start of the study (reference category is the 12-39-year-old age group); $age_{2,i}$ is similarly defined for those aged 40-64 years old; $age_{3,i}$ represents those aged ≥65 years old; and $cases_t$ describes the time-varying risk of infection from the community and is computed as the 7-day moving average of the logarithm of the number of positive PCR tests in the data (which we standardized during model fitting by subtracting the mean and dividing by the standard deviation) (Fig. S1).

Similarly, the individual- and time-specific risk of transmission from an infectious individual *j* to a susceptible household member *i* is defined (for *j≠i*) as:

$$\ln\left(p_{ijt}\right) = \alpha_0 + \beta_1 vax_{1,it} + \beta_2 vax_{2,it} + \beta_3 vax_{3,it} + \kappa_1 vax_{1,jt} + \kappa_2 vax_{2,jt} + \kappa_3 vax_{3,jt} + \gamma_1{}^{HH} * age_{1,i} + \gamma_2{}^{HH} * age_{2,i} + \gamma_3{}^{HH} * age_{3,i} \qquad (3)$$

where α_0 is the baseline risk of infection from an infected household member, and $vax_{v,jt}$ is the vaccination status (for category *v*) of infectious household member *j*; the remaining terms have been previously defined. Note that the age parameters for this model (e.g. $\gamma_1{}^{HH}$) are different from the ones in the community-risk model (e.g. γ_1^{comm}) to account for potential differences in the risk of exposure from an infectious household member versus the community.

To evaluate potential differences in the risk of infection and the vaccine effects following the emergence of the Delta variant, we included interaction terms with a binary indicator θ_t representing time points before ($\theta_t = 0$) and after ($\theta_t = 1$) June 1, 2021. This date was chosen because it corresponds to a period of low incidence prior to the "fourth wave" of cases predominantly associated with the Delta variant in mid-June (Fig. S1). We evaluated models including interactions with the community risk of infection (δ_0), the household risk of infection (α_0), the vaccination status of the susceptible individual *i* and the vaccination status of their contact(s) j. Based on a comparison of the Akaike Information Criterion (AIC) (Figs. S9 and S10), we identified the best-fit model as:

$$\ln(p_{i0t}) = {\delta_0}^{pre} + {\delta_0}^{post}\theta_t + vax_{1it}({\beta_1}^{pre} + {\beta_1}^{post}\theta_t) + vax_{2it}({\beta_2}^{pre} + {\beta_2}^{post}\theta_t) + vax_{3it}({\beta_3}^{pre} + {\beta_3}^{post}\theta_t) + {\gamma_1}^{comm}age_{1,i} + {\gamma_2}^{comm}age_{2,i} + {\gamma_3}^{comm}age_{3,i} + \delta_1 cases_t \quad (4)$$

for the risk of infection for susceptible individual *i* from the community, and

$$\ln(p_{ijt}) = {\alpha_0}^{pre} + {\alpha_0}^{post}\theta_t + vax_{1it}({\beta_1}^{pre} + {\beta_1}^{post}\theta_t) + vax_{2it}({\beta_2}^{pre} + {\beta_2}^{post}\theta_t) + vax_{3it}({\beta_3}^{pre} + {\beta_3}^{post}\theta_t) + vax_{1jt}({\kappa_1}^{pre} + {\kappa_1}^{post}\theta_t) + vax_{2jt}({\kappa_2}^{pre} + {\kappa_2}^{post}\theta_t) + vax_{3jt}({\kappa_3}^{pre} + {\kappa_3}^{post}\theta_t) + {\gamma_1}^{HH}age_{1,i} + {\gamma_2}^{HH}age_{2,i} + {\gamma_3}^{HH}age_{3,i} \quad (5)$$

for the risk of infection for susceptible individual *i* from an infectious household member *j* in household *h*.

The chain binomial model was fit 100 times to the augmented data with different draws from the delay distributions to assess uncertainty in the results due to uncertainty in the unobserved dates of infection and infectiousness. We summarized the parameter estimates by taking the mean over the 100 samples. The law of total variance was used to correctly quantify this uncertainty when constructing 95% confidence intervals (CI) for the parameters of interest. Maximum likelihood analyses were carried out using the *nlm* function [29] within the R statistical software [30].

<u>Vaccine effectiveness</u>

Vaccine effectiveness is expressed as a percentage and computed as 100%*(1-*RR*), with *RR* defined as a risk ratio comparing vaccinated and unvaccinated individuals. For example, vaccine effectiveness against susceptibility to infection between 10-90 days from the second dose during the Delta period is defined as:

$$VE_S = 100\% * \{1 - \exp({\beta_2}^{pre} + {\beta_2}^{post})\},$$

and is based on the vaccination status of individual *i*.

Vaccine effectiveness against infectiousness given infection between 10-90 days from the second dose during the Delta period is defined as:

$$VE_I = 100\% * \{1 - \exp({\kappa_2}^{pre} + {\kappa_2}^{post})\},$$

which is based on the vaccination status of infectious household member *j*.

We also estimated the total vaccine effectiveness between 10-90 days from the second dose during the Delta period as:

$$VE_T = 100\% * \{1 - ({\beta_2}^{pre} + {\beta_2}^{post} + {\kappa_2}^{pre} + {\kappa_2}^{post})\},$$

which correspond to the reduction in risk associated with vaccine-derived protection against both infection of individual *i* and infectiousness given infection for contact *j*. The vaccine effects for different time periods follow similarly.

Secondary attack rate estimation

We estimated the secondary attack rate (SAR) from our model (which accounts for potential tertiary transmission within households and the risk of infection from the community) as:

$$SAR = \sum_{\tau_{infectious}=1}^{\max(\tau_{infectious})} \Pr(\tau_{infectious}) * \{1 - (1 - {\alpha_0}^{pre})^{\tau_{infectious}}\}$$

where α_0 is the baseline probability of transmission per day from an infected HH member to a susceptible adult, $\tau_{infectious}$ is the duration of the infectious period, and $\Pr(\tau_{infectious})$ is its probability density function.

Sensitivity analyses

We conducted sensitivity analyses to test the robustness of the results to the choice of the delay distributions. The delay distributions for the latent period, infectious period, and time from onset of infectiousness to testing were based on estimates from the literature, but may vary over time and/or for our study population. To examine the sensitivity of our results to the parameterization of the delay distributions, we computed the vaccine effectiveness estimates when we increased the variance of the Gamma distributions by a factor of (a) 1.5 and (b) 2, while keeping the same mean (Figs. S2-S4).

The time from onset of infectiousness to testing is not well described in the literature. Previous studies in Israel have assumed that individuals are tested 5-6 days after infection, comparable to the incubation period (time to appearance of symptoms among symptomatic cases), or 1-2 days longer than the latent period (time to onset of infectiousness) [28]. Hence, for our main analysis, we assume a mean reporting delay from onset of infectiousness to testing of 1.5 days, with variance equal to the mean. In sensitivity analyses, we also varied the mean and variance of the reporting delay to be 0 days, 3 days, or 4.5 days (Fig. S5).

Previous household analyses have found no association between age and infectious of individuals [31]. Therefore, we did not include age of the infectious contact as a predictor in our main analysis. However, age of the infectious individual could confound estimates of the VE_I. We therefore performed a sensitivity analysis to determine if including age of infectious contacts (<12 years old versus ≥12 years old) would alter our estimates of VE_I. We set the reference category to be the age group ≥12 years old and estimated the parameters corresponding to the vaccine effects for 10 samples from the delay distributions. Even though we found a small association between age and infectiousness (*RR*=0.90, 95% CI: [0.85, 0.94]), the vaccine effects did not change significantly (Table S10).

Simulation analysis

To validate our model, we simulated infections occurring in a population with households of varying sizes (1, 2 or 3 household members) for a total of 600,000 individuals. We assumed 50% of individuals were vaccinated; the true vaccine effectiveness against susceptibility to infection was $VE_{S,true}$= 90%, while the vaccine effectiveness against infectiousness given infection was $VE_{I,true}$= 50%. Days of infectiousness and positive tests were also simulated for infected individuals by sampling from the delay distributions. We generated 20 independent simulated datasets, and for each dataset, we estimated the parameters corresponding to the vaccine effects using our chain binomial model for 10 samples from the delay distributions. Model code is available from Github [24].

We found that the $VE_{S,true}$ fell within the 95% CI of the summary estimate 85% of the time (17 out of 20 simulated datasets), but the potential bias in estimates of the VE_S was small (<1%) (Table S5). The $VE_{I,true}$ fell within the 95% CI of the summary estimate 100% of the time, but the confidence intervals were wide and some of the point estimates were >10% lower than the true value. The 95% CIs from a single imputation of the missing data contained the true value of the $VE_{S,true}$ and $VE_{I,true}$ 85% (170 out of 200) and 89% (188 out of 200) of the time, respectively.

Additionally, we used the simulated data to test the robustness of the results to misclassification of cases who were not tested. To examine the potential impact of misclassification of individuals who were infected but did not receive a SARS-CoV-2 test, we explored different scenarios based on the probability of detection of cases, both per-day and overall, while assuming that the unidentified cases follow the same infection process as the identified cases. In the first case, we assessed the robustness of the results when decreasing the daily probability of detection *p_detect_day* for any given case in the simulated dataset during their infectious period. We set *p_detect_day* as (a) 0.90 and (b) 0.75 (Table S6). Alternatively, as a second analysis, we assigned each infected case an overall probability *p_no_test* of not getting tested, and therefore detected, and changed their infection status to 0 for all days. Again, we explored values of *p_no_test* as (a) 0.10 or (b) 0.25 (Table S7). In all instances, the 95% confidence interval of the estimated vaccine effects contained the “true” vaccine effectiveness used to generate the simulated data. Results refer to a single realization of the simulated data.

Figure S1. Time series of positive PCR tests during the study period, i.e. June 1, 2020 to July 28, 2021. Left panel: The number of positive PCR tests per day is plotted in black and the 7-day weekly moving average is plotted in blue. Right panel: standardized 7-day weekly moving average of the logarithm of the positive PCR tests. The red dotted vertical line represents the start of the vaccination campaign (December 20, 2020), while the black dotted vertical line represents the start of the Delta period (June 1, 2021).

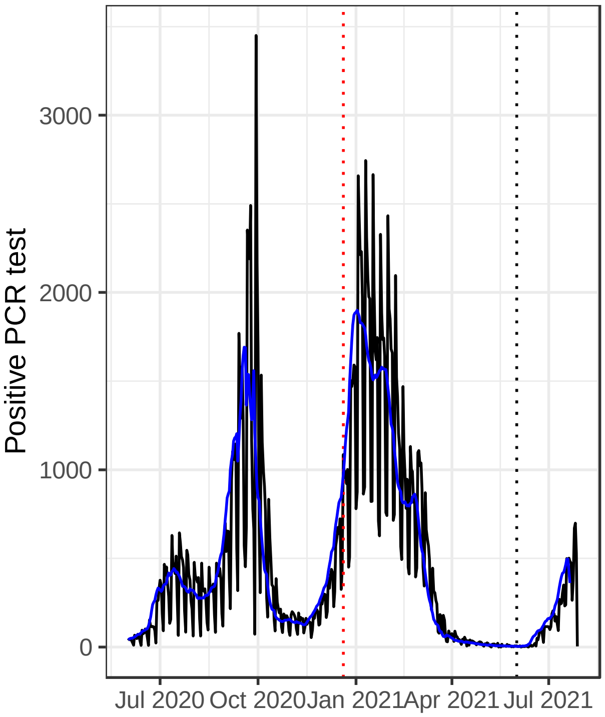

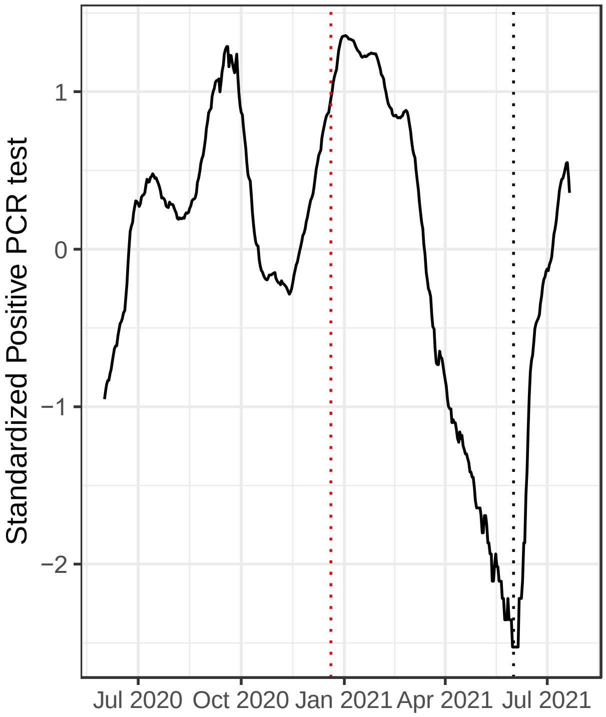

Figure S2. Vaccine effectiveness against susceptibility to infection averaged over 100 iterations of the delay distributions when the variances of the delay distributions are increased by a factor of 1.5 and of 2. (A) VE_{S1} between 10 days from the first dose until 10 days after the second dose; (B) VE_{S2} between 10-90 days from the second dose; and (C) VE_{S3} for ≥90 days from the second dose in the pre-Delta period. Panels D-F correspond to the same time-since-vaccination (VE_{Sv}) estimates for the post-Delta period. Results from the main analysis ("main") are plotted in blue, while results when increasing the variances of the delay distributions by a factor **1.5** ("sens1") and by a factor **2** ("sens2") are plotted in black. Vertical lines correspond to the 95% CIs from all imputations based on the law of total variance.

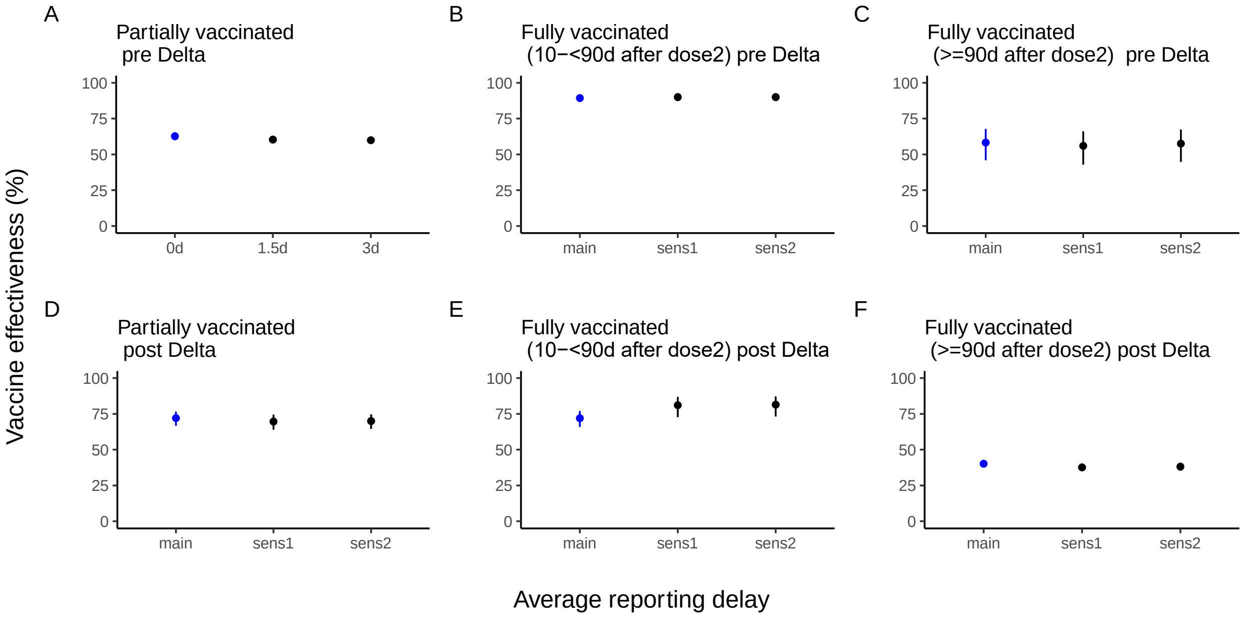

Figure S3. Vaccine effectiveness against infectiousness given infection averaged over 100 iterations of the delay distributions when the variances of the delay distributions are increased by a factor of 1.5 and of 2. (A) VE_{I1} between 10 days from the first dose until 10 days after the second dose; (B) VE_{I2} between 10-90 days from the second dose; and (C) VE_{I3} for ≥90 days from the second dose in the pre-Delta period. Panels D-F correspond to the same time-since-vaccination (VE_{Iv}) estimates for the post-Delta period. Results from the main analysis ("main") are plotted in blue, while results when increasing the variances of the delay distributions by a factor **1.5** ("sens1") and by a factor **2** ("sens2") are plotted in black. Vertical lines correspond to the 95% CIs from all imputations based on the law of total variance.

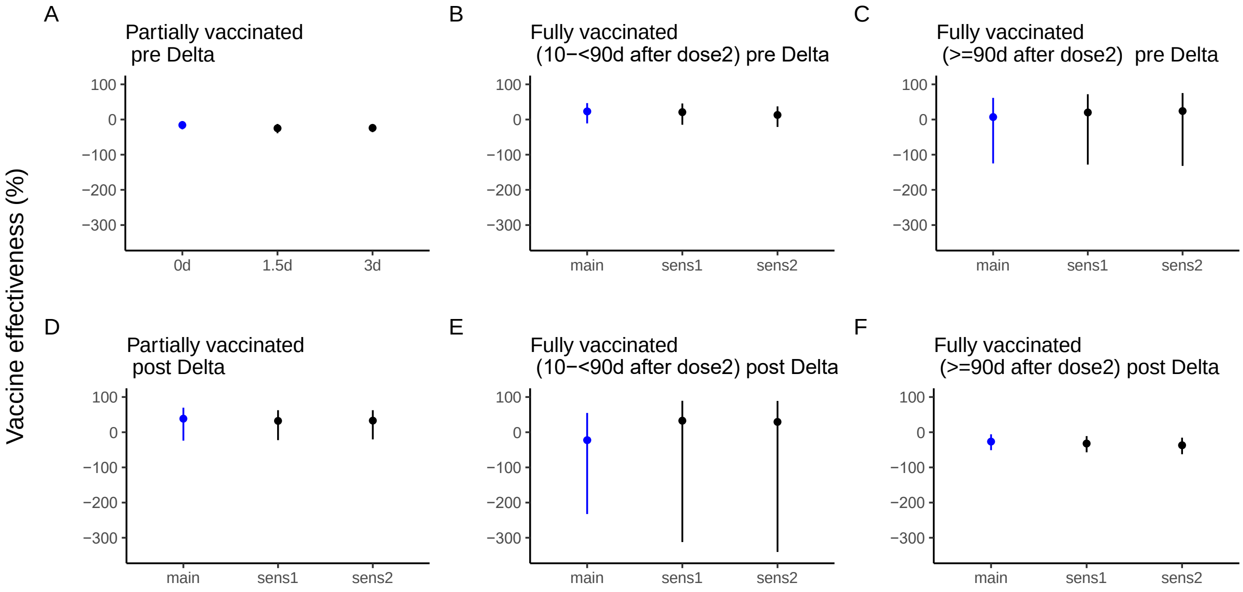

Figure S4. Total vaccine effectiveness averaged over 100 iterations of the delay distributions when the variances of the delay distributions are increased by a factor of 1.5 and of 2. (A) VE_{T1} between 10 days from the first dose until 10 days after the second dose; (B) VE_{T2} between 10-90 days from the second dose; and (C) VE_{T3} for ≥90 days from the second dose in the pre-Delta period. Panels D-F correspond to the same time-since-vaccination (VE_{Tv}) estimates for the post-Delta period. Results from the main analysis ("main") are plotted in blue, while results when increasing the variances of the delay distributions by a factor **1.5** ("sens1") and by a factor **2** ("sens2") are plotted in black. Vertical lines correspond to the 95% CIs from all imputations based on the law of total variance.

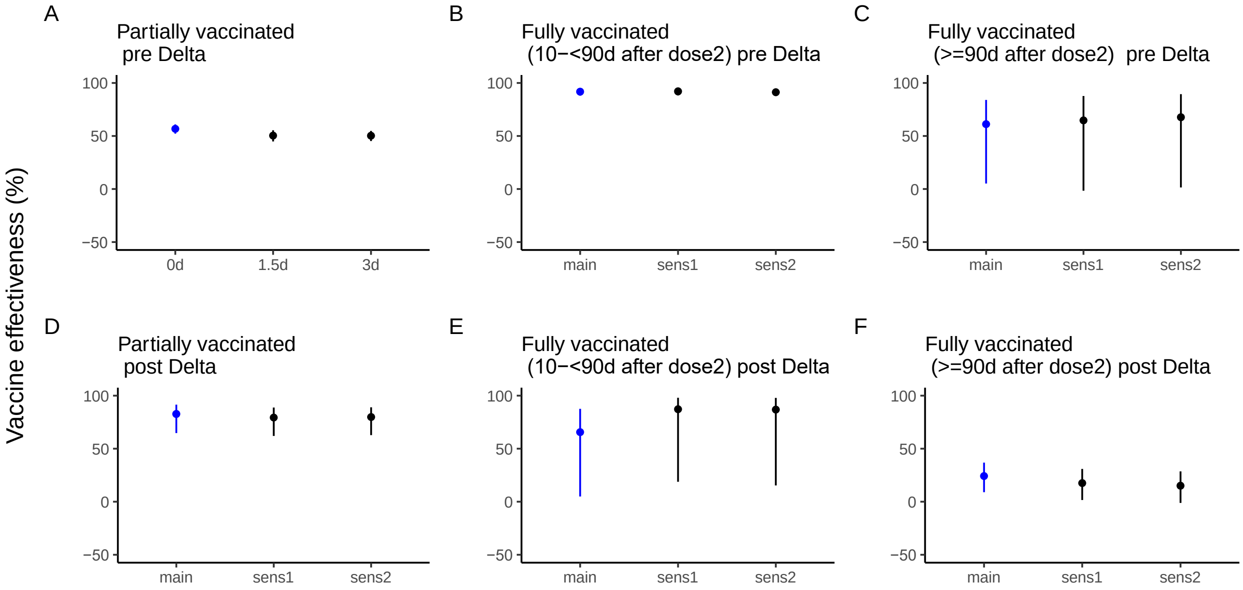

Figure S5. Vaccine effectiveness against infectiousness given infection averaged over 100 iterations of the delay distributions when the mean of the time from onset of infectiousness to testing is increased from 0 to 4.5 days. (A) VE_{I1} between 10 days from the first dose until 10 days after the second dose; (B) VE_{I2} between 10-90 days from the second dose; and (C) VE_{I3} for ≥90 days from the second dose in the pre-Delta period. Panels D-F correspond to the same time-since-vaccination (VE_{Iv}) estimates for the post-Delta period. Results from the main analysis with a mean reporting delay of 1.5 days are plotted in blue, while results for sensitivity analyses assuming the mean reporting delay is 0, 3, or 4.5 days are shown in black. Vertical lines correspond to the 95% CIs from all imputations based on the law of total variance.

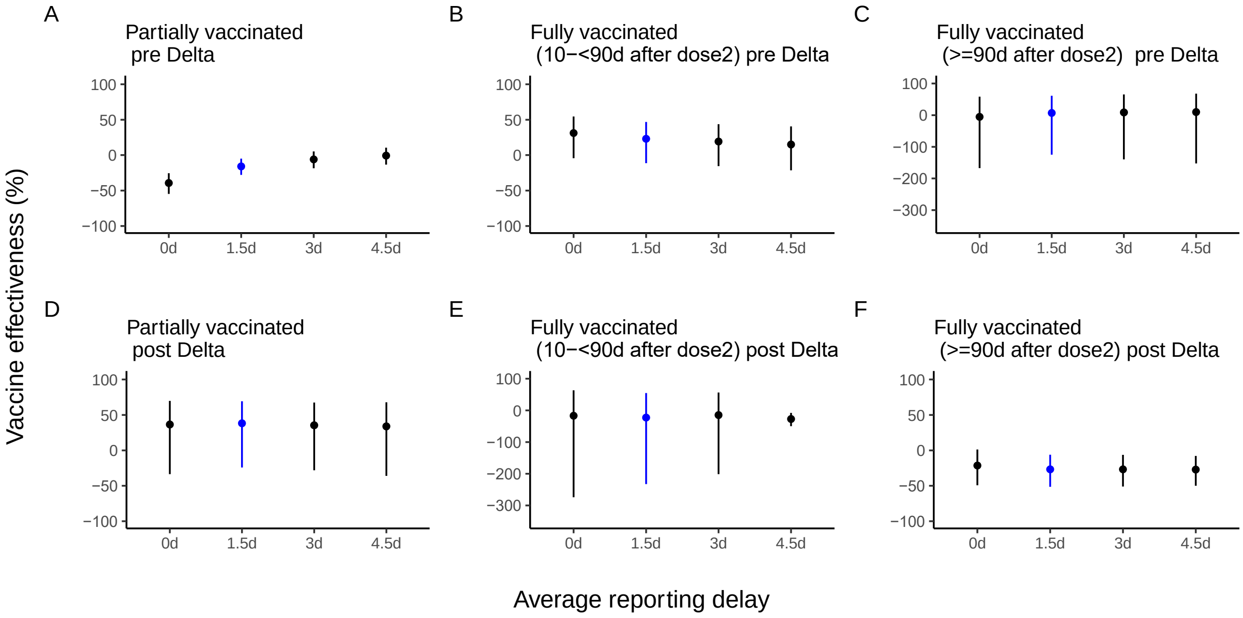

Figure S6. Forest plots of the vaccine effectiveness against susceptibility to infection estimates across the 100 iterations of the delay distributions. (A) VE_{S1} between 10 days from the first dose until 10 days after the second dose; (B) VE_{S2} between 10-90 days from the second dose; and (C) VE_{S3} for ≥90 days from the second dose in the pre-Delta period. Panels D-F correspond to the same time-since-vaccination (VE_{Sv}) estimates for the post-Delta period. The red lines at the bottom of each plot represent the summarized vaccine effectiveness estimates from the 100 iterations of the delay distributions. Horizontal bars correspond to 95% CIs.

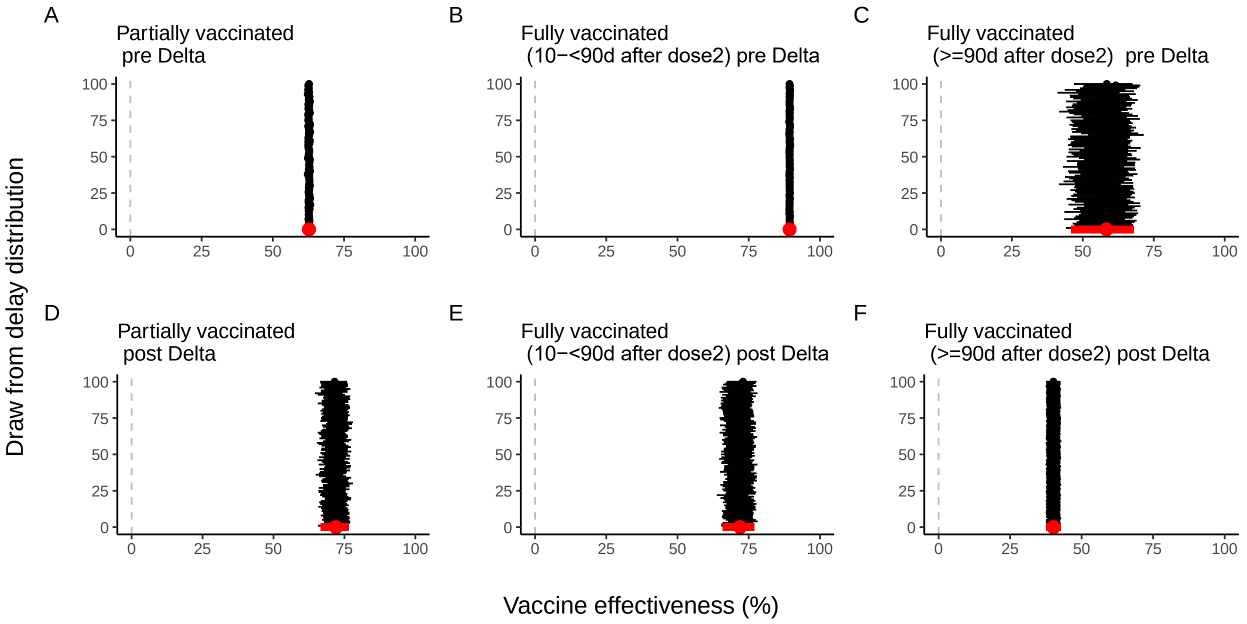

Figure S7. Forest plots of the vaccine effectiveness against infectiousness given infection estimates across the 100 iterations of the delay distributions. (A) VE_{I1} between 10 days from the first dose until 10 days after the second dose; (B) VE_{I2} between 10-90 days from the second dose; and (C) VE_{I3} for ≥90 days from the second dose in the pre-Delta period. Panels D-F correspond to the same time-since-vaccination (VE_{Iv}) estimates for the post-Delta period. The red lines at the bottom of each plot represent the summarized vaccine effectiveness estimates from the 100 iterations of the delay distributions. Horizontal bars correspond to 95% CIs.

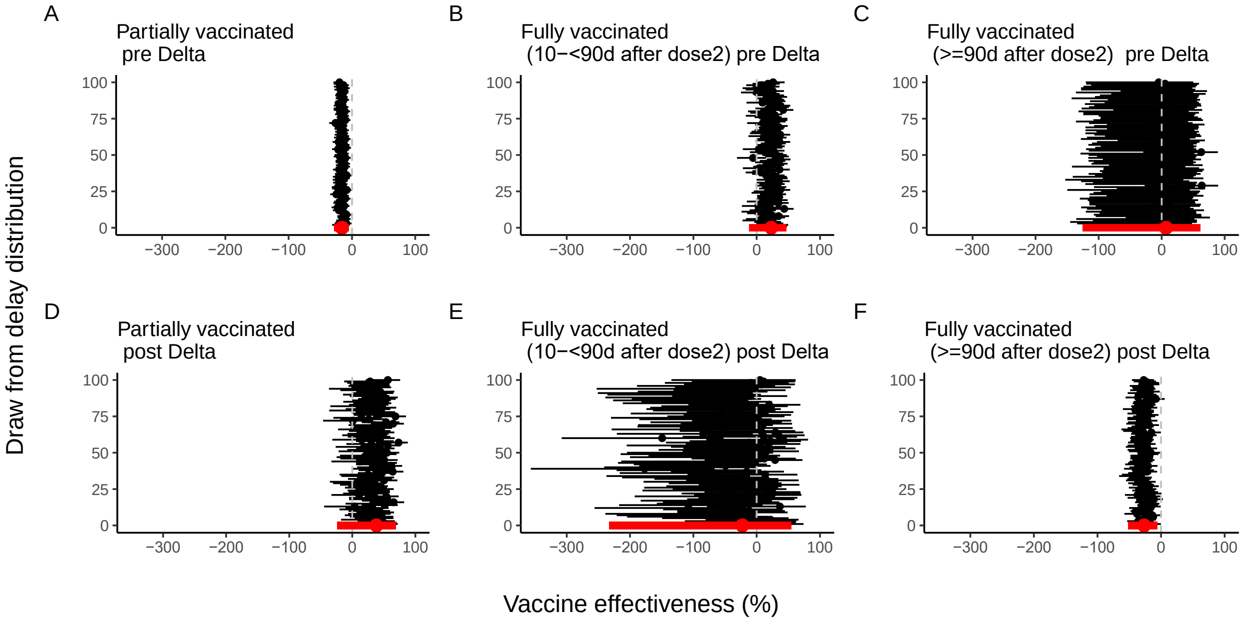

Figure S8. Forest plots of the total vaccine effectiveness estimates across the 100 iterations of the delay distributions. (A) VE_{T1} between 10 days from the first dose until 10 days after the second dose; (B) VE_{T2} between 10-90 days from the second dose; and (C) VE_{T3} for ≥90 days from the second dose in the pre-Delta period. Panels D-F correspond to the same time-since-vaccination (VE_{Tv}) estimates for the post-Delta period. The red lines at the bottom of each plot represent the summarized vaccine effectiveness estimates from the 100 iterations of the delay distributions. Horizontal bars correspond to 95% CIs.

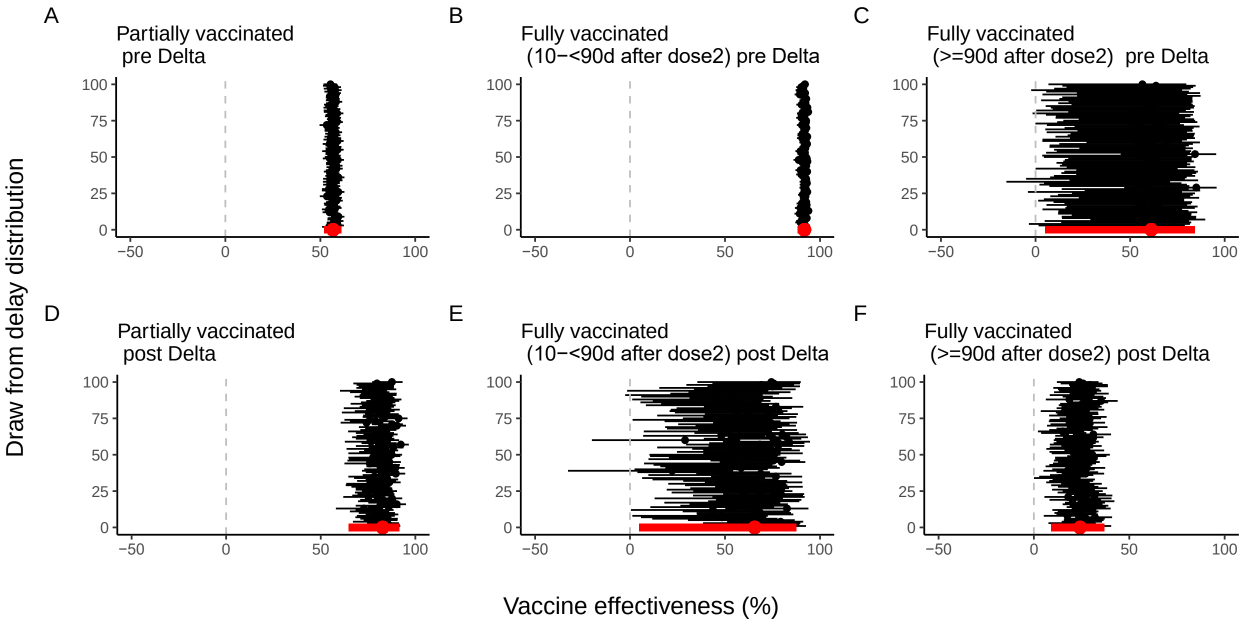

Figure S9. Comparison of models with and without interaction terms for the pre- and post-Delta period. Histogram showing the difference in Akaike Information Criteria (AIC) between model 1, which does not include interaction terms with a binary indicator for before/after June 1, 2021, as opposed to a model with all interactions included (model 2) for 100 iterations of the delay distributions. Results show model 2 (including all interaction terms) is preferred.

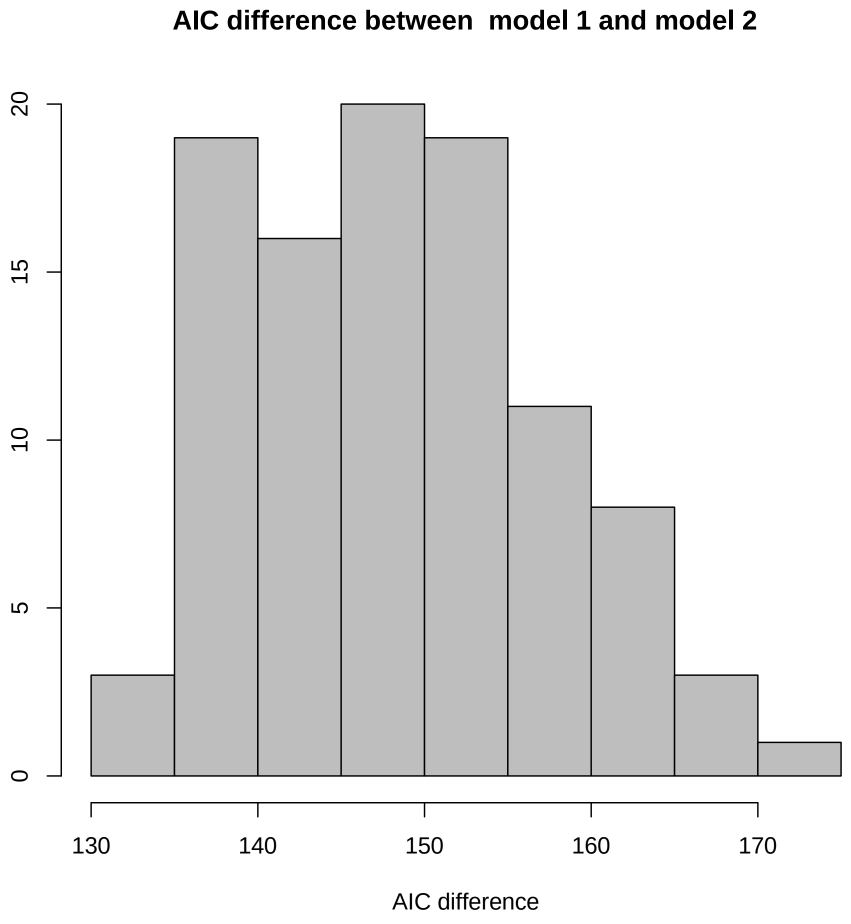

Figure S10. Comparison of models with and without interaction terms for the vaccine effectiveness against infectiousness in the pre- and post-Delta period. Histogram showing the difference in AIC between model 2, i.e. all interactions included with the binary indicator for before/after June 1, 2021, and a model with no interaction terms for the vaccine effects of the contacts (i.e. assuming the vaccine effectiveness against infectiousness is the same pre- and post-Delta periods) (model 3) for 100 iterations of the delay distributions. Results show model 2 (including all interaction terms) is slightly preferred for most iterations of the delay distributions.

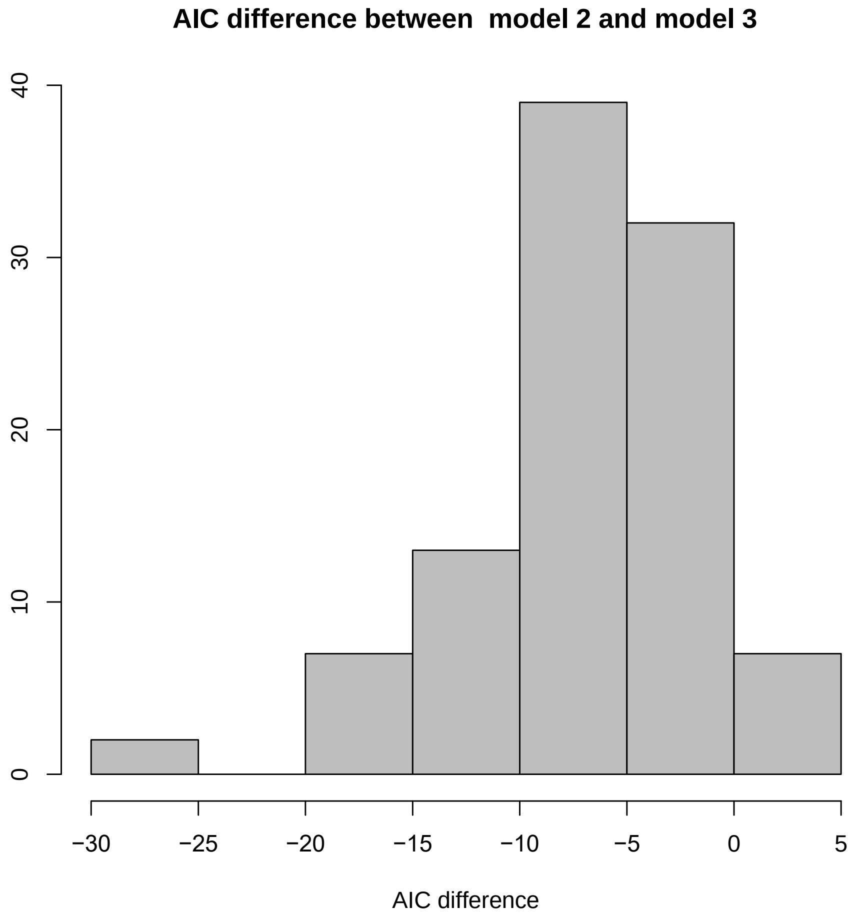

Table S1. Characteristics of the individuals in the study according to their age and sex between June 1, 2020 and July 28, 2021.

**Characteristics**	Fully vaccinated* (n=758,228)	Partially vaccinated* (n=105,305)	Unvaccinated (n=1,608,969)	Overall (n=2,472,502)
**Age years, mean (SD)**	48.3 (18.9)	39.8 (18.6)	27.4 (21.8)	34.3 (23.0)
**Sex – no. (%)**				
Female	377,420 (49.8%)	49,442 (47.0%)	783,280 (48.7)	1,210,142 (48.9)
Male	380,808 (50.2%)	55,863 (53.0%)	825,689 (51.3%)	1,262,360 (51.1%)
**Age group – no. (%)**				
0 to 11 yr	1 (0.0%)	9 (0.0%)	517,773 (32.2%)	517,783 (20.9%)
12 to 39 yr	261,977 (34.6%)	54,765 (52.0%)	623,723 (38.8%)	940,465 (38.0%)
40 to 64 yr	263,748 (34.8%)	33,716 (32.0%)	309,748 (19.3%)	607,212 (24.6%)
≥65 yr	232,502 (30.7%)	16,815 (16.0%)	157,725 (9.8%)	407,042 (16.5%)
**Ever had a SARS-CoV-2 positive PCR test**				
No	751,145 (99.1%)	96,465 (91.6%)	1,421,994 (88.4%)	2,270,204 (91.8%)
Yes	6483 (0.9%)	8840 (8.4%)	186,975 (11.6%)	202,298 (8.2%)

SD – Standard Deviation; yr – year; (*) Individuals are defined as partially or fully vaccinated based on whether 10 days had elapsed since they had received the first/second dose of vaccine at the time of their positive PCR test date, or if no positive PCR test occurred, the end of follow-up.

Table S2. Distribution of PCR-confirmed SARS-CoV-2 infections across households of varying size.

Number of infected household members	Number of household members					
	1	2	3	4	5+	Total (%)
0	734875	221812	95195	77018	65153	1194053 (89.9)
1	62295	13211	8041	6856	8482	98885 (7.4)
2		8151	3706	3042	3492	18391 (1.4)
3			2802	2218	2507	7527 (0.6)
4				1939	2299	4238 (0.3)
5+					4553	4553 (0.3)
**Total (%)**	797170 (60.0)	243174 (18.3)	109744 (8.3)	91073 (6.9)	86486 (6.5)	1327647

Table S3. Naïve secondary attack rates (SAR) according to the vaccination status of the index case, i.e. the first person to test positive in each household. We only included infected contacts who tested positive within 14 days of the “index case”. We excluded data from 7,951 households in which there was more than one index case.

Index	Infected contacts/Total contacts	SAR - % pre Delta	Infected contacts/Total contacts	SAR - % post Delta
Unvaccinated	49413/184191	26.8	1084/5586	19.4
Partially vaccinated - (≥10d dose 1 and <10d dose 2)	1822/6131	29.7	39/257	15.2
Fully vaccinated - (≥10d dose 2 and <90d dose 2)	119/861	13.8	13/57	22.8
Fully vaccinated ((≥90d dose 2)	10/72	13.9	813/3377	24.1

Table S4. Delay distribution parameters for the data augmentation process.

Parameter	Description	Value	Reference
$\boldsymbol{\tau_{report}}$	distribution for the time from onset of infectiousness to testing	~ Gamma(shape=1.5; scale=1)	[28]
$\boldsymbol{\tau_{infectious}}$	distribution for the infectious period	~ Gamma(shape=4; scale=5/4)	[27]
$\boldsymbol{\tau_{latent}}$	distribution for the latent period	~ Gamma(shape=4; scale=1)	[27]

Table S5. Vaccine effectiveness estimates using simulated data. Estimates of vaccine effectiveness against susceptibility to infection (VE_S) and against infectiousness given infection (VE_I) when using simulated data (with $VE_{S,true}$=90% and $VE_{I,true}$=50%). Estimates from 20 simulated datasets are presented, with 95% confidence intervals (CI) computed using the law of total variance from 10 imputed datasets each. The second and fourth columns report the number of CIs for each imputed dataset that contain the true value, while the final row reports the proportion of summary 95% CIs that contain the true value.

Dataset	VE_S [95%CI] (True value=90%)	Number of simulations with true value	VE_I [95%CI] (True value=50%)	Number of simulations with true value
1	89.6% [89.2%, 90.1%]	10/10	49.0% [23.6%, 65.9%]	10/10
2	89.5% [89.0%, 90.0%]	9/10	55.1% [32.0%, 70.4%]	10/10
3	90.0% [89.5%, 90.5%]	10/10	35.4% [7.5%, 54.9%]	5/10
4	90.2% [89.7%, 90.7%]	10/10	29.8% [0.7%, 50.4%]	5/10
5	90.1% [89.7%, 90.6%]	10/10	56.8% [30.7%, 73.1%]	10/10
6	89.9% [89.4%, 90.4%]	10/10	45.4% [16.5%, 64.4%]	10/10
7	90.2% [89.8%, 90.7%]	10/10	47.6% [13.6%,68.2%]	10/10
8	90.1% [89.6%, 90.6%]	10/10	35.5% [9.2%, 54.2%]	7/10
9	89.3% [88.9%, 89.8%]	0/10	48.8% [26.2%, 64.5%]	10/10
10	88.9% [88.4%, 89.4%]	0/10	39.9% [10.5%, 59.6%]	8/10
11	89.7% [89.2%, 90.2%]	10/10	45.1% [19.8%, 62.5%]	9/10
12	89.9% [89.4%, 90.4%]	10/10	41.4% [11.3%, 61.3%]	8/10
13	90.0% [89.6%, 90.5%]	10/10	48.2% [24.7%, 64.3%]	10/10
14	89.2% [88.7%, 89.7%]	0/10	37.3% [8.0%, 57.2%]	7/10
15	89.9% [89.4%, 90.4%]	10/10	44.7% [13.9%, 64.6%]	9/10
16	89.8% [89.3%, 90.3%]	10/10	52.1% [28.2%, 68.0%]	10/10
17	89.9% [89.5%, 90.4%]	10/10	49.6% [25.7%, 65.8%]	10/10
18	89.9% [89.4%, 90.4%]	10/10	40.5% [14.0%, 58.8%]	9/10
19	90.0% [89.5%, 90.5%]	10/10	44.7% [17.4%, 63.0%]	10/10
20	89.5% [89.0%, 90.0%]	10/10	56.9% [35.4%, 71.2%]	10/10
**TOTAL**		17/20		20/20

Table S6. Vaccine effectiveness estimates for varying probability of detection using simulated data. Estimates of the vaccine effectiveness against susceptibility to infection and against infectiousness given infection when we assume the probability of detection of an infected individual is 0.90 or 0.75 on each day of their infectious period for simulated data.

True VE	P.detect.day=0.90	P.detect.day=0.75
Vaccine effectiveness against susceptibility to infection (True value=90%)		
90%	89.4% [88.9%, 89.9%]	89.9% [89.4%, 90.5%]
Vaccine effectiveness against infectiousness given infection (True value=50%)		
50%	55.0% [31.4%, 70.4%]	36.0% [0.4%, 58.9%]

Table S7. Vaccine effectiveness estimates for misclassified infections using simulated data. Estimates of vaccine effectiveness against susceptibility to infection and against infectiousness given infection when we assume the probability of an infected individual not getting tested (i.e. being misclassified) is 0.10 or 0.25.

True VE	P.no.test=0.10	P.no.test=0.25
Vaccine effectiveness against susceptibility to infection (True value=90%		
90%	89.6% [89.1%, 90.1%]	89.7% [89.1%, 90.3%]
Vaccine effectiveness against infectiousness given infection (True values=50%)		
50%	51.9% [25.7%, 68.9%]	41.8% [5.8%, 64.0%]

Table S8. Vaccine effectiveness against infectiousness given infection (VE_I) of household contacts when restricting the data to unvaccinated susceptible children <12 years of age.

Type of vaccine effectiveness measure	Time since vaccination	Estimate pre-Delta [95% confidence interval]	Estimate post-Delta [95% confidence interval]
Vaccine effectiveness against infectiousness given infection			
VE_{I1}	>10d dose 1 and <10d dose 2	-25.3% [-46.1%, -7.4%]	57.0% [-40.8%, 86.9%]
VE_{I2}	≥10d dose 2 and <90d dose 2	41.0% [-13.7%, 69.4%]	-91.2% [-706.6%, 54.7%]
VE_{I3}	≥90d dose 2	15.1% [-17671.7%, 99.6%]	-7.1% [-40.3%, 18.3%]

d – days.

Table S9. Description of the parameter estimates on the relative risk (RR) scale with 95% confidence intervals (CI).

Parameter	Description	RR [95%CI]
$\exp(\boldsymbol{\alpha_0^{pre}})$	baseline probability of transmission per day from an infected HH member to a susceptible adult (i.e., ref category) – pre-Delta	0.021 [0.020, 0.021]
$\exp(\boldsymbol{\delta_0^{pre}})$	baseline probability of transmission per day from the community to a susceptible adult (i.e., ref category) – pre-Delta	0.00018 [0.00017, 0.00018]
$\exp(\boldsymbol{\alpha_0^{post}})$	baseline probability of transmission per day from the household - post-Delta	0.93 [0.84, 1.04]
$\exp(\boldsymbol{\delta_0^{post}})$	baseline probability of transmission per day from the community - post-Delta	1.13 [1.09, 1.16]
$\exp(\boldsymbol{\beta_1}^{pre})$	vaccination status of the individual (10d from dose 1 to <10d from dose 2) pre-Delta	0.37 [0.36, 0.38]
$\exp(\boldsymbol{\beta_2}^{pre})$	vaccination status of the individual (10 to 89 days from dose 2) pre-Delta	0.11 [0.10, 0.11]
$\exp(\boldsymbol{\beta_3}^{pre})$	vaccination status of the individual (≥ 90 days from dose 2) pre-Delta	0.42 [0.32, 0.54]
$\exp(\boldsymbol{\beta_1}^{post})$	vaccination status of the individual post-Delta (10d from dose 1 to <10d from dose 2)	0.75 [0.62, 0.90]
$\exp(\boldsymbol{\beta_2}^{post})$	vaccination status of the individual post-Delta (10 to 89 days from dose 2)	2.64 [2.15, 3.23]
$\exp(\boldsymbol{\beta_3}^{post})$	vaccination status of the individual post-Delta (≥ 90 days from dose 2)	1.43 [1.10, 1.87]
$\exp(\boldsymbol{\kappa_1}^{pre})$	vaccination status of other HH members (10d from dose 1 to <10d from dose 2) pre-Delta	1.16 [1.05, 1.28]
$\exp(\boldsymbol{\kappa_2}^{pre})$	vaccination status of other HH members (10 to 89 days from dose 2) pre-Delta	0.77 [0.53, 1.11]
$\exp(\boldsymbol{\kappa_3}^{pre})$	vaccination status of other HH members (≥ 90 days from dose 2) pre-Delta	0.93 [0.39, 2.24]
$\exp(\boldsymbol{\kappa_1}^{post})$	vaccination status of other HH members (10d from dose 1 to <10d from dose 2) post-Delta	0.53 [0.26, 1.08]
$\exp(\boldsymbol{\kappa_2}^{post})$	vaccination status of other HH members (10 to 89 days from dose 2) post-Delta	1.59 [0.56, 4.52]
$\exp(\boldsymbol{\kappa_3}^{post})$	vaccination status of other HH members (≥ 90 days from dose 2) post-Delta	1.36 [0.55, 3.34]
$\exp(\boldsymbol{\gamma_1^{comm}})$	age <12 (community specific)	0.50 [0.50, 0.51]
$\exp(\boldsymbol{\gamma_2}^{comm})$	age ≥40 and <64 (community specific)	0.77 [0.76, 0.78]
$\exp(\boldsymbol{\gamma_3}^{comm})$	ages ≥ 65 (community specific)	0.52 [0.51, 0.53]
$\exp(\boldsymbol{\gamma_1^{HH}})$	age <12 (household specific)	0.74 [0.72, 0.77]
$\exp(\boldsymbol{\gamma_2}^{HH})$	age ≥40 and <64 (household specific)	1.07 [1.03, 1.12]
$\exp(\boldsymbol{\gamma_3}^{HH})$	ages ≥ 65 (household specific)	1.84 [1.67, 2.02]
$\exp(\boldsymbol{\delta_1})$	time-varying risk from the community*	4.05 [4.00, 4.11]

d– days; *relative risk for each 1 SD increase in the average number of cases in the previous 7-day period.

Table S10. Vaccine effectiveness against infectiousness given infection (VE_I) of household contacts when distinguishing between the age of the infectious contact, i.e. children (<12 years of age) and adults (≥12 years of age).

Type of vaccine effectiveness measure	Time since vaccination	Estimate pre-Delta [95% confidence interval]	Estimate post-Delta [95% confidence interval]
Vaccine effectiveness against infectiousness given infection			
VE_{I1}	>10d dose 1 and <10d dose 2	-14.4% [-26.8%, -3.1%]	43.5% [-38.1%, 76.9%]
VE_{I2}	≥10d dose 2 and <90d dose 2	17.4% [-12.5%, 39.3%]	-4.0% [-134.4%, 53.8%]
VE_{I3}	≥90d dose 2	0.1% [-129.3%, 56.4%]	-21.7% [-44.3%, -2.7%]

d – days.
