# Supplementary material for: Vaccination with BNT162b2 reduces transmission of SARS-CoV-2 to household contacts in Israel
Source: Science. 2022 Jan 27;375(6585):eabl4292. doi: 10.1126/science.abl4292 (PMC9261115; doi:10.1126/science.abl4292)
Supplement: Supplementary file 2 — MDAR Reproducibility Checklist [file science.abl4292_mdar_reproducibility_checklist.pdf]

Materials Design Analysis Reporting (MDAR) Checklist for Authors

The MDAR framework establishes a minimum set of requirements in transparent reporting applicable to studies in the life sciences (see Statement of Task: doi:10.31222/osf.io/9sm4x.). The MDAR checklist is a tool for authors, editors and others seeking to adopt the MDAR framework for transparent reporting in manuscripts and other outputs. Please refer to the MDAR Elaboration Document for additional context for the MDAR framework.

Materials

Antibodies	Yes (indicate where provided: page no/section/legend)	n/a
For commercial reagents, provide supplier name, catalogue number and RRID, if available.		X

Cell materials	Yes (indicate where provided: page no/section/legend)	n/a
**Cell lines:** Provide species information, strain. Provide accession number in repository **OR** supplier name, catalog number, clone number, **OR** RRID		X
**Primary cultures:** Provide species, strain, sex of origin, genetic modification status.		X

Experimental animals	Yes (indicate where provided: page no/section/legend)	n/a
**Laboratory animals:** Provide species, strain, sex, age, genetic modification status. Provide accession number in repository **OR** supplier name, catalog number, clone number, **OR** RRID		X
**Animal observed in or captured from the field:** Provide species, sex and age where possible		X
**Model organisms:** Provide Accession number in repository (where relevant) **OR** RRID		X

Plants and microbes	Yes (indicate where provided: page no/section/legend)	n/a
**Plants:** provide species and strain, unique accession number if available, and source (including location for collected wild specimens)		X
**Microbes:** provide species and strain, unique accession number if available, and source		X

Human research participants	Yes (indicate where provided: page no/section/legend)	n/a
Identify authority granting ethics approval (IRB or equivalent committee(s), provide reference number for approval.	Page 8, section “Data and materials availability ”	
Provide statement confirming informed consent obtained from study participants.		X
Report on age and sex for all study participants.		X

Design

**Study protocol**	**Yes (indicate where provided: page no/section/legend)**	**n/a**
For clinical trials, provide the trial registration number **OR** cite DOI in manuscript.		X

**Laboratory protocol**	**Yes (indicate where provided: page no/section/legend)**	**n/a**
Provide DOI or other citation details if detailed step-by-step protocols are available.		X

**Experimental study design (statistics details)**	**Yes (indicate where provided: page no/section/legend)**	**n/a**
State whether and how the following have been done**, or** if they were not carried out.		
Sample size determination		X
Randomisation		X
Blinding		X
Inclusion/exclusion criteria		X

**Sample definition and in-laboratory replication**	**Yes (indicate where provided: page no/section/legend)**	**n/a**
State number of times the experiment was replicated in laboratory		X
Define whether data describe technical or biological replicates		X

**Ethics**	**Yes (indicate where provided: page no/section/legend)**	**n/a**
Studies involving human participants: State details of authority granting ethics approval (IRB or equivalent committee(s), provide reference number for approval.		X
Studies involving experimental animals: State details of authority granting ethics approval (IRB or equivalent committee(s), provide reference number for approval.		X
Studies involving specimen and field samples: State if relevant permits obtained, provide details of authority approving study; if none were required, explain why.		X

**Dual Use Research of Concern (DURC)**	**Yes (indicate where provided: page no/section/legend)**	**n/a**
If study is subject to dual use research of concern, state the authority granting approval and reference number for the regulatory approval		X

Analysis

Attrition	Yes (indicate where provided: page no/section/legend)	n/a
State if sample or data point from the analysis is excluded, and whether the criteria for exclusion were determined and specified in advance.	Page 10, "Data sources" section in the SM	

Statistics	Yes (indicate where provided: page no/section/legend)	n/a
Describe statistical tests used and justify choice of tests.	Methods section in the SM	

Data Availability	Yes (indicate where provided: page no/section/legend)	n/a
State whether newly created datasets are available, including protocols for access or restriction on access.		X
If data are publicly available, provide accession number in repository or DOI or URL.		X
If publicly available data are reused, provide accession number in repository or DOI or URL, where possible.		X

Code Availability	Yes (indicate where provided: page no/section/legend)	n/a
For all newly generated code and software essential for replicating the main findings of the study:		
State whether the code or software is available.	Yes	
If code is publicly available, provide accession number in repository, or DOI or URL.	10.5281/zenodo.5670699	

Reporting

Adherence to community standards	Yes (indicate where provided: page no/section/legend)	n/a
MDAR framework recommends adoption of discipline-specific guidelines, established and endorsed through community initiatives. Journals have their own policy about requiring specific guidelines and recommendations to complement MDAR.		X
State if relevant guidelines (eg., ICMJE, MIBBI, ARRIVE) have been followed, and whether a checklist (eg., CONSORT, PRISMA, ARRIVE) is provided with the manuscript.		X
