# Supplementary material for: Vaccination with BNT162b2 reduces transmission of SARS-CoV-2 to household contacts in Israel
Source: Science. Author manuscript; Available in PMC 2022 Sep 11. (PMC9261115; doi:10.1126/science.abl4292)
Supplement: Supplementary file 2 [file NIHMS1817170-supplement-Table_S1__updated_.docx]

**Table** **S1.** **Characteristics of the individuals in the study according to their age and sex between June 1, 2020 and July 28, 2021****.** 

| **Characteristics** | Fully vaccinated* (n=1,457,912) | Partially vaccinated* (n=44,564) | Unvaccinated (n=970,026) | Overall (n=2,472,502) |
| --- | --- | --- | --- | --- |
| **Age years, mean (SD)** | 44.6 (18.9) | 29.7 (19.7) | 19.2 (20.0) | 34.3 (23.0) |
| **Sex – no. (%)** |  |  |  |  |
| Female | 699,753 (48.0%) | 22,250 (49.9%) | 488,139 (50.3%) | 1,210,142 (48.9%) |
| Male | 758,159 (52.0%) | 22,314 (50.1%) | 481,887 (49.7%) | 1,262,360 (51.1%) |
| **Age group – no. (%)** |  |  |  |  |
| 0 to 11 yr | 6 (0.0%) | 19 (0.0%) | 517,758 (53.4%) | 517,783 (20.9%) |
| 12 to 39 yr | 618,990 (42.5%) | 31,974 (71.7%) | 289,501 (29.8%) | 940,465 (38.0%) |
| 40 to 64 yr | 493,973 (33.9%) | 7,943 (17.8%) | 105,296 (10.9%) | 607,212 (24.6%) |
| ≥65 yr | 344,943 (23.7%) | 4,628 (10.4%) | 57,471 (5.9%) | 407,042 (16.5%) |
| **Ever had a SARS-CoV-2 positive PCR test** |  |  |  |  |
| No | 1,451,429 (99.6%) | 35,724 (80.2%) | 783,051 (80.7%) | 2,270,204 (91.8%) |
| Yes | 6483 (0.4%) | 8840 (19.8%) | 186,975 (19.3%) | 202,298 (8.2%) |
| **Person-time (days)** | 211,412,210 | 33,906,217 | 794,343,238 | 1,039,661,665 |

SD – Standard Deviation; yr – years; (*) Individuals are defined as partially or fully vaccinated based on whether 10 days had elapsed since they had received the first/second dose of vaccine at the time of their positive PCR test date, or if no positive PCR test occurred, the end of follow-up.
